# Metabolic Stress Expands Polyfunctional, Proinflammatory Th_17_ Cells in Patients With Psoriatic Arthritis for Whom There is Interleukin‐23–Independent Interleukin‐17 Production

**DOI:** 10.1002/art.43095

**Published:** 2025-02-08

**Authors:** Carmel B. Stober, Louise Ellis, Jane C. Goodall, Marc Veldhoen, J. S. Hill Gaston

**Affiliations:** ^1^ University of Cambridge and Addenbrooke's Hospital, Cambridge University Hospitals NHS Foundation Trust Cambridge United Kingdom; ^2^ University of Cambridge, Cambridge University Hospitals NHS Foundation Trust Cambridge United Kingdom; ^3^ GIMM ‐ Gulbenkian Institute for Molecular Medicine, Avenida Prof. Egas Moniz Lisbon Portugal; ^4^ Faculdade de Medicina da Universidade de Lisboa Avenida Prof. Egas Moniz Lisbon Portugal; ^5^ The Babraham Institute Cambridge United Kingdom

## Abstract

**Objective:**

Genetic associations and blockade of the interleukin (IL)‐23/IL‐17 axis with monoclonal antibodies support a role for this pathway in patients with psoriatic arthritis (PsA). This study examines the requirement of IL‐23 for IL‐17 production and the role of the metabolic microenvironment in the expansion of Th_17_‐derived cells in patients with PsA.

**Methods:**

Th_17_ cell frequencies in synovial fluid or peripheral blood from patients with PsA were evaluated by flow cytometry using chemokine receptor 6, CD161, and T‐bet as phenotypic markers, and the cytokines interferon γ, granulocyte–macrophage colony‐stimulating factor (GM‐CSF), and IL‐17 were assessed by flow cytometry and enzyme‐linked immunosorbent assay. The impact of IL‐23 and metabolic stress on T cell differentiation was investigated.

**Results:**

Polyfunctional positive IL‐17 (IL‐17^pos^) CD4 (*P* < 0.0001) and CD8 (*P* < 0.0001), and GM‐CSF^pos^ Th_17_‐derived cells (*P* < 0.0001) were increased in the inflamed joints of patients with PsA, with a proportional decrease in the peripheral blood of patients. We demonstrate IL‐23–independent IL‐17 release by CD4 T cells in patients with PsA, in which the absence of IL‐23 during Th_17_ differentiation reduced IL‐17 by mean ± SEM 31% ± 5.8%. Exogenous IL‐23 increased IL‐17, negatively regulated GM‐CSF, and cooperated with transforming growth factor β to augment IL‐17. Polyfunctional Th_17_ and Th_17_‐derived cells, but not Th_1_ cells, were expanded by metabolic stress in patients with PsA.

**Conclusion:**

We confirmed the abundance of polyfunctional type 17 CD4 and CD8 cells in the inflamed joints of patients with PsA. We demonstrate IL‐23–independent expansion of Th_17_ cells, for which IL‐23 negatively regulates GM‐CSF. This may account for therapeutic differences in IL‐17 and IL‐23 inhibition in patients with PsA or other spondyloarthritides. Polyfunctional IL‐17^pos^ Th_17_ and Th_17_‐derived but not Th_1_ cells were expanded by metabolic stress, and metabolic stress may itself represent a unique therapeutic target.

## INTRODUCTION

Psoriasis (Ps) is a common chronic inflammatory skin condition affecting 1% to 3% of the world population,^1^ and up to 30% of patients with Ps have psoriatic arthritis (PsA).^2^ Genetic studies have highlighted the importance of the interleukin (IL)‐23 pathway, with a unique PsA risk variant identified at the IL‐23 receptor (IL‐23R) locus.^3^, ^4^ The clinical importance of the IL‐23/Th_17_ pathway in patients with PsA has also been validated by the efficacy of antibodies targeting IL‐23 or IL‐17. We showed elevated frequencies of Th_17_ cells in the synovial fluid (SF) of patients with PsA,^5^, ^6^ with more recent evidence revealing that these T cells are associated with polyfunctional cytokine expression.^7^, ^8^, ^9^ However, the benefit of therapies targeting the IL‐23/Th_17_ pathway are more impressive for patients with Ps than for patients with PsA, and IL‐23 inhibition has been ineffective in patients with axial disease.^10^ This implies differences in tissue‐specific effector cells and the possibility that cytokines in addition to IL‐17 contribute to pathogenicity in patients with PsA.

Granulocyte–macrophage colony‐stimulating factor (GM‐CSF) is a hematopoietic growth factor that promotes the maturation and activation of monocytes, dendritic cells, and neutrophils and enhances the release of pro‐inflammatory cytokines. In experimental autoimmune encephalomyelitis (EAE), an autoimmune mouse model of multiple sclerosis (MS), GM‐CSF produced by Th_17_ was essential for disease,^11^, ^12^, ^13^ whereas IL‐17A and IL‐17F were not required.^14^ In these models, pathogenic Th_17_‐derived or ex‐Th_17_ cells possessed features of Th_17_ but no longer produced IL‐17; they coexpressed the Th_1_‐associated transcription factor T‐bet in addition to retinoic acid receptor–related orphan nuclear receptor (ROR) γt, but also secreted GM‐CSF, and were expanded by IL‐23.^15^, ^16^, ^17^ GM‐CSF–producing T cells have been observed at sites of inflammation in human diseases including cerebrospinal fluid from patients with MS,^18^, ^19^ SF from patients with rheumatoid arthritis (RA) and juvenile idiopathic arthritis,^20^ and peripheral blood (PB) from patients with spondyloarthritis.^7^

The role of IL‐23 in Th_17_ cell differentiation has remained controversial because naive T cells lack IL‐23R,^21^, ^22^ although transforming growth factor (TGF) β, IL‐1, and IL‐6 induce the expression of the Th_17_ cell–specific transcription factor RORγt, leading to the expression of *Il23r* and *Il17a*.^23^ Naive T cells differentiated with TGFβ and IL‐6 are nonpathogenic,^24^ but exposure to IL‐23 conferred pathogenicity.^25^ It was therefore hypothesized that IL‐6 is absolutely required for Th_17_ cell differentiation,^26^ whereas IL‐23 was necessary for expansion and stabilization of the Th_17_ cell phenotype^22^, ^27^ and the induction of pathogenic Th_17_ and Th_17_‐derived subsets that produce GM‐CSF.^17^, ^22^ A relationship between metabolic stress, IL‐23R expression, and Th_17_ cell development was demonstrated in murine studies, in which exposure of T cells to increased salt concentrations expanded pathogenic Th_17_ cells, up‐regulated pro‐inflammatory GM‐CSF, and accelerated autoimmunity.^28^, ^29^ Moreover, a high‐salt diet in mice and a pilot human study reduced several intestinal bacteria, promoted hypertension, and increased the frequency of Th_17_ cells.^30^

We confirm increased frequencies of polyfunctional type 17 and positive GM‐CSF (GM‐CSF^pos^) Th_17_‐derived cells in inflamed joints, with a relative reduction of these subsets in the PB of patients with PsA. We demonstrate IL‐23‐independent IL‐17 release by Th_17_ cells, and in contrast to murine studies, IL‐23 negatively regulates GM‐CSF. We identify metabolic stress as a putative target for intervention in patients with PsA, in which inhibiting glycolysis reduces IL‐17, whereas ionic and endoplasmic reticular (ER) stress expand polyfunctional Th_17_ and Th_17_‐derived cells but not Th_1_ cells in patients with PsA.

## PATIENTS AND METHODS

### Patient samples

PB (n = 60) and SF (n = 10) samples were obtained from patients with PsA attending the rheumatology service at Addenbrooke's Hospital who fulfilled Criteria of the Classification of Psoriatic Arthritis criteria.^31^ Table 1 summarizes the demographics of patients with PsA recruited to the study, including erythrocyte sedimentation rate and C‐reactive protein values, number of tender or swollen joints, and the proportion of patients receiving biologic, synthetic, or no disease‐modifying antirheumatic drugs. Blood was taken from healthy donor (HD) volunteers (n = 24). The study was approved by the Addenbrooke's Hospital and Repatriation General Hospital local ethics committees.

**Table 1:** Demographic and clinical characteristics of patients with PsA*

Characteristic	All PsA (n = 60), median (IQR)	SF (n = 10), median (IQR)	PsA vs SF, *P* value	HD (n = 24), median (IQR)	PsA vs HD, *P* value
Male, n (%)	37 (62)	8 (80)	0.21	17 (71)	0.41
Age, y	48 (40–58)	53 (43–68)	0.33	40 (32–48)	**0.05**
ESR, mm/h	8 (5–19)	13 (7–39)	0.17	–	–
CRP, ng/mL	4 (4–14)	20 (4–41)	0.09	–	–
68 TJC	2 (1–5)	1 (1–2)	0.42	–	–
66 SJC	1 (0–3)	1 (1–2)	0.98	–	–
Treatment, n (%)					
Biologic ± synthetic DMARD	13 (22)	0	–	–	–
Synthetic DMARD	25 (42)	6 (60)	–	–	–
No DMARD	22 (37)	4 (40)	–	–	–

* 60 patients with psoriatic arthritis (PsA) were recruited to this study, including 10 patients who provided synovial fluid (SF). Bold *P* values are considered statistically significant. CRP, C‐reactive protein; DMARD, disease‐modifying antirheumatic drug; ESR, erythrocyte sedimentation rate; HD, healthy donor; IQR, interquartile range; SJC, swollen joint count; TJC, tender joint count.

### Cell isolation

SF mononuclear cells (SFMCs) and peripheral blood mononuclear cells (PBMCs) were purified by density centrifugation.^5^ Naive CD4 T cells were isolated using the naive CD4 T cell isolation kit II human (Miltenyi Biotec; Supplementary Figure 1).

### Cell stimulation

PBMCs and SFMCs were treated with phorbol 12‐myristate 13‐acetate (PMA), ionomycin, and BD GolgiStop (BD Biosciences).^5^ Alternatively, cells were incubated with Dynabeads human T‐activator CD3/28 beads (ratio of bead to cell of 1:1) ± IL‐23 or IL‐12 at 10 ng/mL or IL‐2 at 100 U/mL and supernatants were harvested at 72 hours for the quantification of GM‐CSF, IL‐17, or interferon (IFN) γ by enzyme‐linked immunosorbent assay (ELISA; Ready‐SET‐Go!, eBioscience). Naive T cells were cultured in X‐vivo medium (Lonza) with Th_17_ differentiation: IL‐1β, IL‐6, anti‐IFNγ, ± IL‐23 ± TGFβ; Th_1_ differentiation: IL‐12 ± IL‐23 (BD Pharmingen); and Dynabeads human T‐activator CD3/28 beads. T cell differentiation was also performed in 2 to 10 nM thapsigargin, an additional 50 mM NaCl, or 2 mM 2‐deoxy‐d‐glucose (2‐DG). Supernatants were harvested for ELISA, and cells were restimulated with PMA, ionomycin, and BD GolgiStop before harvesting and staining for flow cytometric analysis.

### Flow cytometry

Cells were stained with Zombie‐NIR, blocked with 1% mouse serum, and stained extracellularly for CD3, CD4, chemokine receptor (CCR) 6, and CD161, and fixed, permeabilized, and stained intracellularly with antibodies to IL‐17, IFNγ, and GM‐CSF (BD Cytofix/Cytoperm) or appropriate isotype controls. For intracellular T‐bet staining, the Transcription Factor Staining buffer set (eBioscience) was used. A Fortessa Flow Cytometry System (BD Biosciences) and FlowJo software (TreeStar) were used for analysis, with assistance provided by the National Institute for Health Research Cambridge Biomedical Research Centre phenotyping hub.

### Statistical analysis

Statistical analysis was performed using Prism 6 software. Two groups were compared using Student's *t*‐test and three or more groups were compared with one‐way analysis of variance and Tukey's multiple comparison test. When the effects of treatment were explored, paired *t*‐tests were used, and *P* <0.05 was considered significant.

## RESULTS

### Polyfunctional GM‐CSF^pos^ and IL‐17^pos^ T cells are abundant in the inflamed joints of patients with PsA and are decreased in PB from patients with PsA compared with PB from HDs

**Figure 1:**
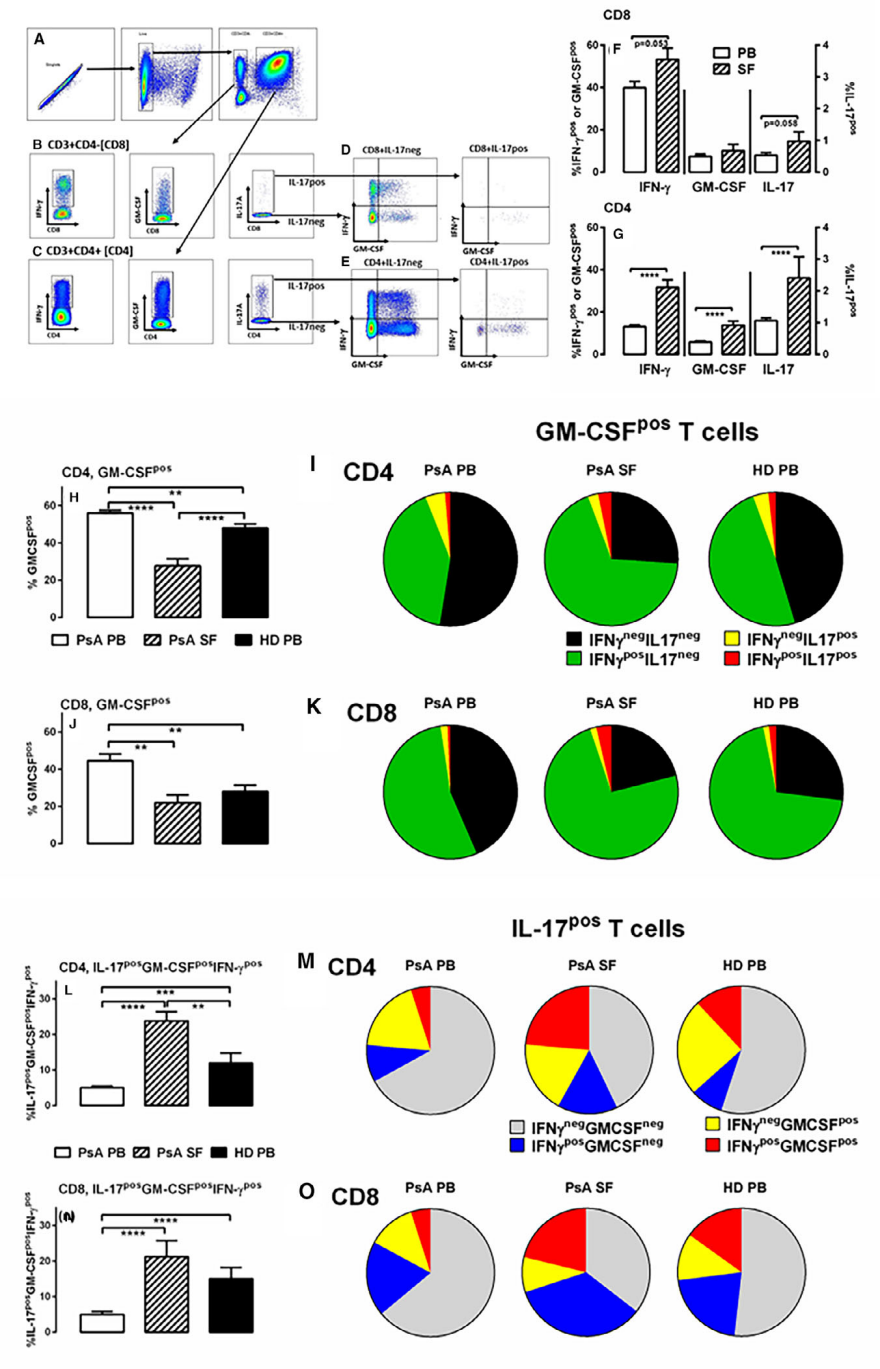
Polyfunctional GM‐CSF^pos^ and IL‐17^pos^ T cells are abundant in inflamed joints of patients with PsA and are decreased in PB of patients with PsA compared with PB from HDs. PB from patients with PsA (n = 60), SF from patients with PsA (n = 10), or PB from HDs (n = 24) were analyzed. PBMCs or SFMCs were stimulated with PMA and ionomycin, before analysis by flow cytometry. (A) PBMCs (or SFMCs) were gated on FSC/SSC to identify lymphocytes, dead cells were excluded, and further gated as (B) CD3+CD4^neg^ (CD8) or (C) CD3+CD4+ (CD4) T cells, expressing either IFNγ, GM‐CSF, or IL‐17. The frequency of (F) CD8 or (G) CD4 T cells that were IFNγ^pos^, GM‐CSF^pos^, or IL‐17^pos^ were compared in PB and SF from patients with PsA. Polyfunctional cytokine expression was then evaluated where (D, upper) CD8 or (E, lower) CD4 T cells were gated as (D and E, left) IL‐17 negative or (D and E, right) IL‐17 positive, and then gated on IFNγ and GM‐CSF expression. (H) CD4 and (J) CD8 were compared in GM‐CSF^pos^ (IFNγ^neg^IL‐17^neg^) subsets, and (L) CD4 and (N) CD8 IL‐17^pos^GM‐CSF^pos^IFNγ^pos^ subsets were compared in PB and SF from patients with PsA and PB from HDs. The coexpression of GM‐CSF with other cytokines in (I) CD4 and (K) CD8 T cells; and the coexpression of IL‐17 with other cytokines in (M) CD4 and (O) CD8 T cells; in PB and SF from patients with PsA and PB from HDs are demonstrated in pie charts. Data are represented as mean ± SEM and are analyzed by one‐way ANOVA with Tukey's multiple comparison test. *P < 0.05; **P < 0.01; ***P < 0.001; ****P < 0.0001. ANOVA, analysis of variance; FSC, forward scatter; GM‐CSF, granulocyte–macrophage colony‐stimulating factor; HD, healthy donor; IFN, interferon; IL, interleukin; neg, negative; PB, peripheral blood; PBMC, PB mononuclear cells; PMA, phorbol 12‐myristate 13‐acetate; pos, positive; PsA, psoriatic arthritis; SF, synovial fluid; SFMC, SF mononuclear cells; SSC, side scatter.

The frequency of SF and PB CD4 and CD3+CD4^neg^ (mostly CD8) T cells producing GM‐CSF, IFNγ, or IL‐17 were evaluated in patients with PsA (Figure 1 A–C). IFNγ, GM‐CSF, and IL‐17 were higher in inflamed joints relative to PB from patients, although they were only significant for CD4 T cells (Figure 1G, *P* < 0.0001). GM‐CSF has defined pathogenicity of Th_17_ cells;^11^, ^12^, ^13^, ^15^, ^16^, ^17^ therefore, GM‐CSF coexpression was investigated (Figure 1D and E).

GM‐CSF^pos^ polyfunctional T cells were abundant in inflamed joints, where mean ± SEM 74% ± 4.2% CD4 (Figure 1I) and mean ± SEM 79% ± 4.2% CD8 (Figure 1K) T cells coexpressed GM‐CSF with another cytokine. This was reflected by reduced CD4 (Figure 1H, *P* < 0.001) and CD8 (Figure 1J, *P* < 0.01) T cells from the SF of patients with PsA expressing only GM‐CSF when compared with PB from patients with PsA (n = 60), also confirmed using matched patient SF and PB CD4 (Supplementary Figure 2E, n = 4) and CD8 (Supplementary Figure 2D, n = 4) T cells. GM‐CSF^pos^ polyfunctional T cells were increased in PB from HDs compared with PB from patients with PsA, and observed for CD4 (Figure 1H and I) and CD8 (Figure 1J and K) T cells. Because only 0.7% to 4.8% of GM‐CSF^pos^ cells were IL‐17^pos^ (Figure 1I and K), the expression of IL‐17 with other cytokines was evaluated.

Polyfunctional IL‐17^pos^ T cells were increased in SF from patients with PsA relative to PB from patients with PsA, and observed for CD4 (Figure 1M; mean ± SEM 57% ± 4.6% SF from patients with PsA vs mean ± SEM 33% ± 2.1% PB from patients with PsA; *P* < 0.0001) and CD8 (Figure 1O; mean ± SEM 65% ± 7.1% SF from patients with PsA vs mean ± SEM 36% ± 3.3% PB from patients with PsA; *P* < 0.0001) T cells. Polyfunctional IL‐17^pos^GM‐CSF^pos^IFNγ^pos^ CD4 (Figure 1L, *P* < 0.0001) and CD8 (Figure 1N, *P* < 0.0001) were abundant in SF compared with PB, also confirmed using matched patient SF and PB CD4 (Supplementary Figure 2G, n = 4) and CD8 (Supplementary Figure 2F, n = 4) T cells. Polyfunctional IL‐17^pos^ cells were less abundant in patients with PsA compared with PB from HDs; the highest proportions of IL‐17^pos^GM‐CSF^pos^IFNγ^pos^ CD4 (Figure 1L) and CD8 (Figure 1N) T cells were observed in SF from patients with PsA, which is greater than from PB from HDs, which in turn is greater than from PB from patients with PsA. We therefore confirm accumulation of polyfunctional GM‐CSF^pos^ and IL‐17^pos^ CD4 and CD8 T cells in inflamed joints, with a relative reduction in patients with PsA compared with PB from HDs.

### GM‐CSF^pos^ and IL‐17^pos^ polyfunctional CD4 T cells express Th_17_‐associated phenotypic markers and T‐bet

**Figure 2:**
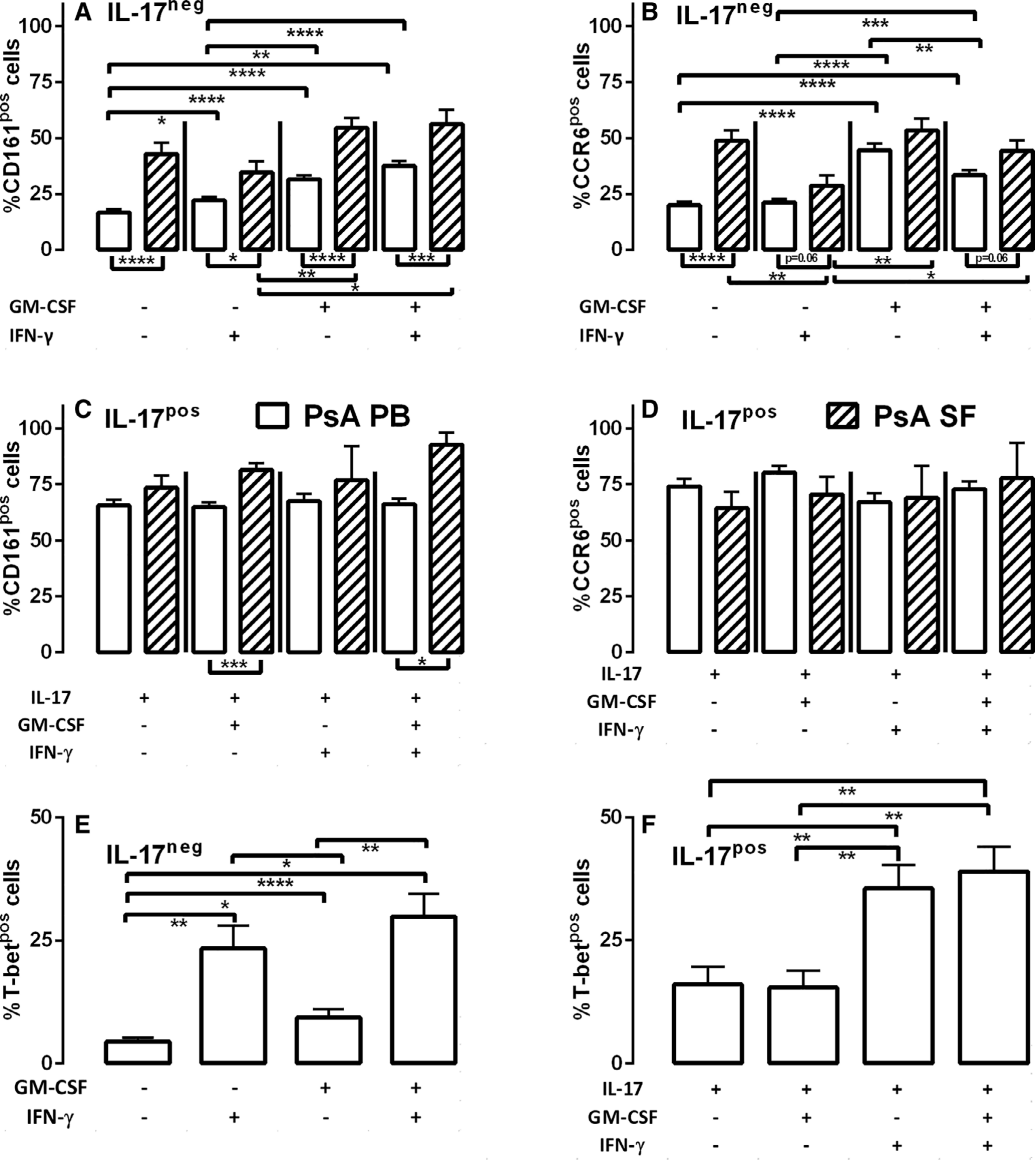
GM‐CSF^pos^ and IL‐17^pos^ polyfunctional CD4 T cells express Th_17_‐associated phenotypic markers and T‐bet. Cells of PB from patients with PsA (n = 38, open bars) and SF from patients with PsA (n = 9, hatched bars) were incubated with PMA and ionomycin before staining and analysis by flow cytometry. Cells were gated as CD3+CD4+ T cells and evaluated as (A and C) CD161^pos^ or (B and D) CCR6^pos^ cells according to cytokine expression profiles (as in Figure 1E). Alternatively, PB from patients with PsA (n = 10) was stimulated with PMA and ionomycin, gated as CD3+CD4+ T cells, and examined based upon cytokine and T‐bet expression. (E and F) T‐bet expression in (E) IL‐17^neg^ or (F) IL‐17^pos^ CD4 T cells coexpressing GM‐CSF ± IFNγ. Data are represented as mean ± SEM, and analyzed by one‐way ANOVA with Tukey's multiple comparison test. *P < 0.05; **P < 0.01; ***P < 0.001; ****P < 0.0001. ANOVA, analysis of variance; CCR, chemokine receptor; GM‐CSF, granulocyte–macrophage colony‐stimulating factor; HD, healthy donor; IFN, interferon; IL, interleukin; neg, negative; PB, peripheral blood; PMA, phorbol 12‐myristate 13‐acetate; pos, positive; PsA, psoriatic arthritis; SF, synovial fluid.

The expression of Th_17_‐associated phenotypic markers was evaluated in inflamed joints and PB from patients with PsA. CD161, a cell‐surface C‐type lectin‐like receptor closely associated with Th_17_ and the transcription factor RORγt, was most abundant in IL‐17^pos^ PB subsets (Figure 2C). The Th_17_‐associated CCR6 was also highly expressed by IL‐17^pos^ Th_17_ cells (Figure 2D), with no significant difference in either CD161 (Figure 2C) or CCR6 (Figure 2D) if IL‐17^pos^ PB cells coexpressed other cytokines. IFNγ^pos^ PB cells not producing GM‐CSF or IL‐17 (likely classic Th_1_) displayed the lowest surface levels of CD161 (Figure 2A) and CCR6 (Figure 2B). GM‐CSF expression correlated with intermediate levels of CD161, significantly higher than for classic Th_1_ (Figure 2A, *P* < 0.01) but lower than IL‐17^pos^ PB cells (Figure 2C, *P* < 0.0001). Intermediate expression levels of CCR6 were also observed in cells expressing GM‐CSF without IL‐17 (Figure 2B).

In inflamed joints, mean ± SEM 43% ± 5.1% and 49% ± 4.7% of cytokine‐negative cells expressed CD161 (Figure 2A) and CCR6 (Figure 2B), respectively, which is higher than equivalent PB T cells. GM‐CSF^pos^ SF T cells demonstrated increased CD161 expression relative to GM‐CSF^pos^ PB subsets, and significantly higher than SF Th_1_ cells (Figure 2A). GM‐CSF expression by IL‐17^pos^ SF T cells also coincided with higher CD161 levels compared with PB counterparts (Figure 2C). CCR6 expression was lower in Th_1_ SF cells when compared with cytokine‐negative and GM‐CSF^pos^ SF T cells (Figure 2B).

The pathogenicity of Th_17_‐derived cells, and the switch of Th_17_ to Th_1_ coincides with expression of the Th_1_‐associated transcription factor, T‐bet.^15^, ^16^, ^17^ We demonstrate T‐bet expression correlates with IFNγ, and was higher in GM‐CSF^pos^ cells coexpressing IFNγ (Figure 2E, *P* < 0.001). Similarly, IFNγ coexpression in IL‐17^pos^ cells was associated with increased T‐bet expression (Figure 2F, *P* < 0.01). GM‐CSF^pos^IFNγ^pos^ cells that no longer produce IL‐17 but express Th_17_‐associated markers therefore likely represent Th_17_‐derived subsets, for which T‐bet acquisition in these and IL‐17^pos^GM‐CSF^pos^IFNγ^pos^ polyfunctional T cells may represent a transition from a Th_17_ to Th_1_ phenotype.

### Exogenous IL‐23 increases IL‐17 but inhibits GM‐CSF release by Teff cells in patients with PsA

**Figure 3:**
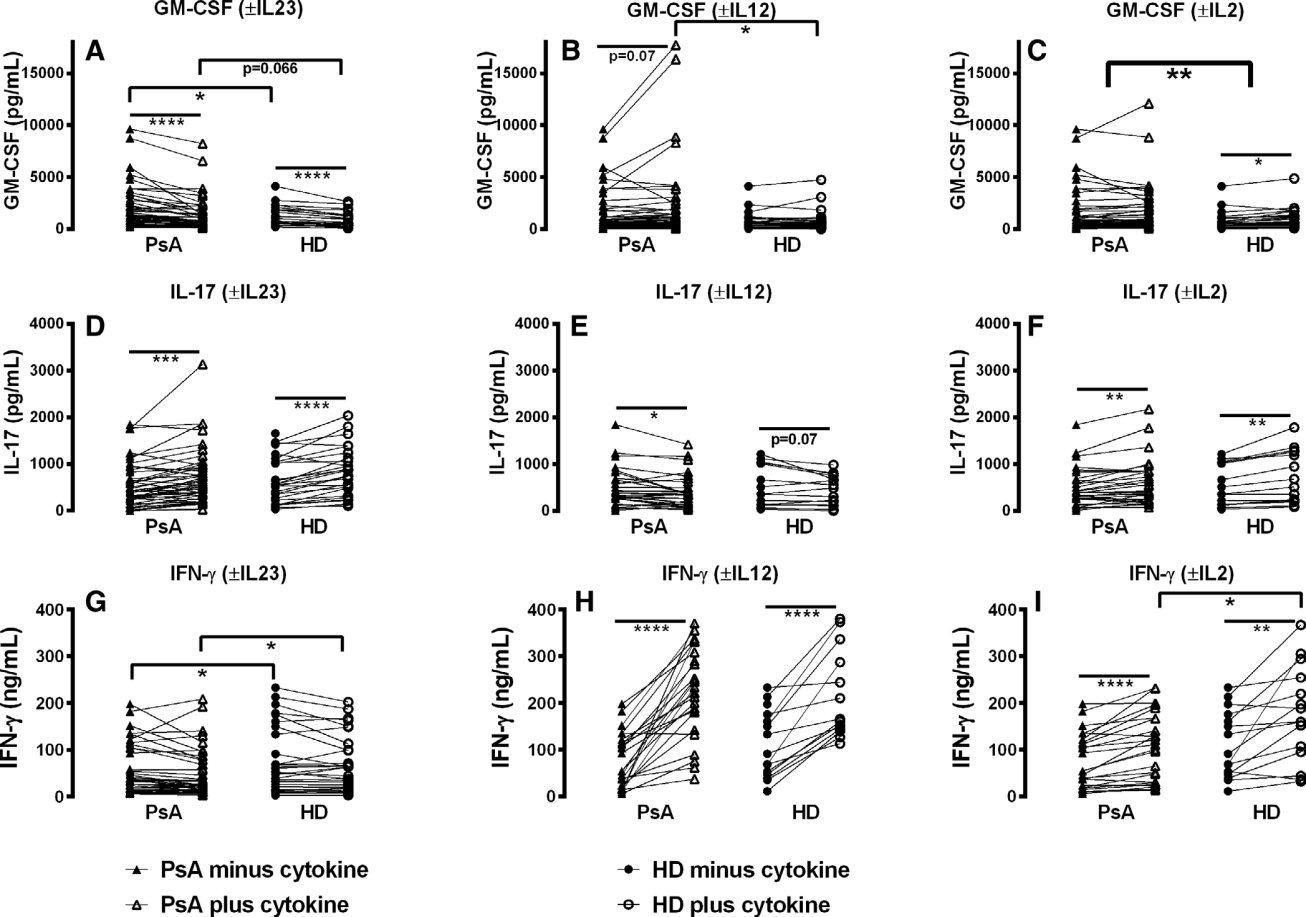
Exogenous IL‐23 increases IL‐17 but inhibits GM‐CSF release by Teff cells in patients with PsA. PBMCs from patients with PsA (n = 50) and HDs (n = 26) were stimulated with anti‐CD3/28 in the absence or presence of exogenous IL‐23, IL‐12, or IL‐2, and supernatants tested for (A–C) GM‐CSF, (D–F) IL‐17, or (G–I) IFNγ by ELISA. Matched pairs of PBMC from patients with PsA (▲) or HDs (●) without exogenous cytokine were compared with PBMCs from patients with PsA (Δ) or HDs (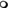) with exogenous IL‐23, IL‐12, or IL‐2. Samples from patients with PsA were also compared with PBMCs from HDs within treatment groups. Data are represented as paired samples, with data analyzed by paired or unpaired Student's *t*‐test. *P < 0.05; **P < 0.01; ***P < 0.001; ****P < 0.0001. ELISA, enzyme‐linked immunosorbent assay; GM‐CSF, granulocyte–macrophage colony‐stimulating factor; HD, healthy donor; IFN, interferon; IL, interleukin; PB, peripheral blood; PBMC, PB mononuclear cell; PsA, psoriatic arthritis.

In mice, IL‐23 expands pathogenic GM‐CSF^pos^ Th_17_ cells;^11^, ^12^, ^13^ therefore, the effect of exogenous IL‐23 on cytokine release was compared in patients with PsA and in HDs. Stimulated Teff cells from patients with PsA released higher GM‐CSF (Figure 3A, *P* < 0.05), lower IFNγ (Figure 3G, *P* < 0.05) and similar levels of IL‐17 (Figure 3D) compared with HDs. In contrast to murine studies, exogenous IL‐23 decreased GM‐CSF release by both patients with PsA (Figure 3A; mean ± SD 31% ± 3.0% reduction; *P* < 0.0001) and HDs (Figure 3A; mean ± SEM 33% ± 4.4% reduction; *P* < 0.001) T cells. IL‐23 increased IL‐17 in patients with PsA (Figure 3D; mean ± SEM 57% ± 13.5% increase; *P* < 0.001) and in HDs (Figure 3D; mean ± SEM 70% ± 15.7% increase; *P* < 0.0001), and there was no net effect of IL‐23 on IFNγ (Figure 3G).

Exogenous IL‐12 augmented IFNγ production (Figure 3H; mean ± SEM 636% ± 150.2% increase; *P* < 0.0001) and slightly increased GM‐CSF in patients with PsA (Figure 3B; mean ± SEM 26% ± 9.0% increase; *P* = 0.07). IL‐17 release was inhibited by IL‐12, although only significant for patients with PsA (Figure 3E, *P* < 0.01).

Polymorphisms in *IL2RA* have been associated with increased frequencies of GM‐CSF‐producing T cells.^18^ We found no net effect of exogenous IL‐2 on GM‐CSF release in patients with PsA, although there was a slight increase in HDs (Figure 3C, *P* < 0.05). IL‐2 enhanced IL‐17 release in patients with PsA and in HDs (Figure 3F; mean ± SEM 39% ± 11.9% increase; *P* < 0.001) and also IFNγ in both patients with PsA (Figure 3I; mean ± SEM 59% ± 12.6% increase; *P* < 0.0001) and HDs (Figure 3I; mean ± SEM 85% ± 26.5% increase; *P* < 0.01).

### IL‐17 release by Th_17_ cells from patients with PsA is only partially IL‐23 dependent, whereas IL‐23 cooperates with TGFβ

We sought to explore the effects of IL‐23 and TGFβ on IL‐17, GM‐CSF, and IFNγ production by naive T cells differentiated under Th_17_‐polarizing or Th_1_‐polarizing conditions, in patients with PsA. Naive CD4 T cells were differentiated with Th_17_‐polarizing cytokines (IL‐1β, IL‐6, anti‐IFNγ) with or without IL‐23 ± TGFβ (Supplementary Figure 3); or the Th_1_‐polarizing cytokine IL‐12 ± IL‐23 (Supplementary Figure 4).

**Figure 4:**
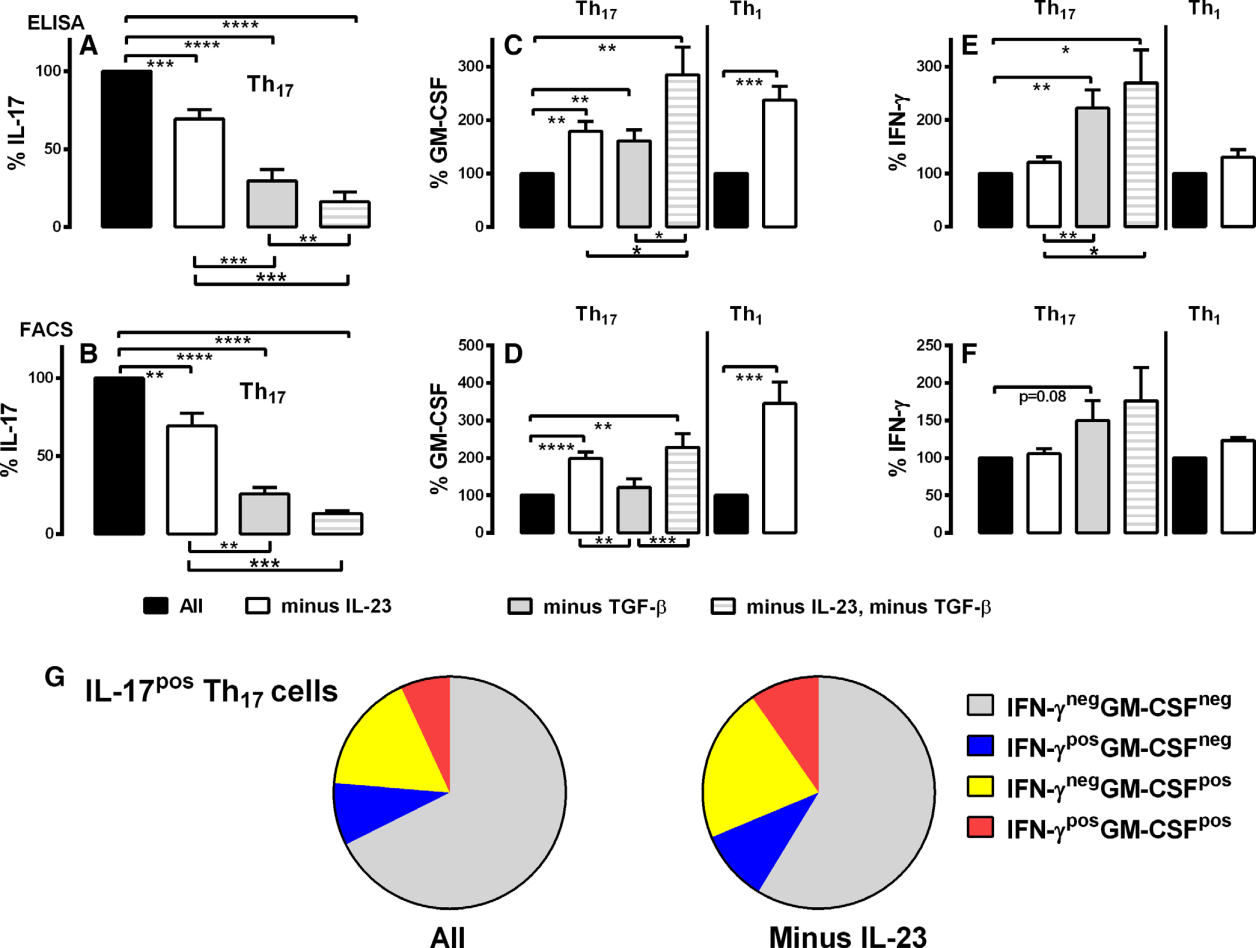
IL‐17 release by Th_17_ cells from patients with PsA is only partially IL‐23 dependent, whereas IL‐23 cooperates with TGFβ. Naive CD4 T cells of patients with PsA were differentiated in (A–G) Th_17_‐polarizing (n = 14) or (C–F) Th_1_‐polarizing (n = 12) conditions with removal of cytokines as detailed and stimulated with anti‐CD3/28 Dynabeads for five to seven days, and supernatants were tested for (A) IL‐17, (C) GM‐CSF, or (E) IFNγ by ELISA. Cells were harvested and restimulated with PMA and ionomycin before staining and analysis by flow cytometry. Cells were gated as live, CD3^+^CD4^+^ T cells, and evaluated as (B) IL‐17^pos^, (D) GM‐CSF^pos^, or (F) IFNγ^pos^ T cells. Data comparing cytokine removal is normalized relative to Th_17_‐polarizing conditions with IL‐23 and TGFβ; or Th_1_ polarizing conditions with IL‐23. (G) Pie charts demonstrating IL‐17^pos^ T cells with the coexpression of GM‐CSF ± IFNγ differentiated under Th_17_ polarizing conditions in the presence or absence of IL‐23. Data are represented as mean ± SEM, with data analyzed by one‐way ANOVA with Tukey's multiple comparison test. *P < 0.05; **P < 0.01; ***P < 0.001; ****P < 0.0001. ANOVA, analysis of variance; ELISA, enzyme‐linked immunosorbent assay; FACS, fluorescence‐activated cell sorting; GM‐CSF, granulocyte–macrophage colony‐stimulating factor; IFN, interferon; IL, interleukin; PMA, phorbol 12‐myristate 13‐acetate; pos, positive; PsA, psoriatic arthritis; TGF, transforming growth factor. Color figure can be viewed in the online issue, which is available at http://onlinelibrary.wiley.com/doi/10.1002/art.43095/abstract.

IL‐23–independent IL‐17 production was confirmed, in that the absence of IL‐23 during Th_17_ differentiation reduced IL‐17 production by mean ± SEM 31% ± 5.8% (Figure 4A; *P* < 0.001), and IL‐17^pos^ T cell frequency by mean ± SEM 31% ± 8.2% (Figure 4B, Supplementary Figure 3A, *P* < 0.01). TGFβ removal during Th_17_ differentiation markedly reduced IL‐17 release by mean ± SEM 70% ± 7.3% (Figure 4A, *P* < 0.0001) and the frequency of IL‐17^pos^ cells by mean ± SEM 74% ± 4.4% (Figure 4B and Supplementary Figure 3A; *P* < 0.0001). IL‐23 and TGFβ cooperated to augment IL‐17 release in that removing both cytokines reduced IL‐17 production by mean ± SEM 84% ± 6.3% (Figure 4A, *P* < 0.0001) and IL‐17^pos^ cells by mean ± SEM 87% ± 2.0% (Figure 4B and Supplementary Figure 3A, *P* < 0.0001).

In contrast to IL‐17, the absence of IL‐23 during Th_17_ differentiation increased GM‐CSF by mean ± SEM 79 ± 19% (Figure 4C; *P* < 0.01), and the frequency of GM‐CSF^pos^ T cells by mean ± SEM 99% ± 17% (Figure 4D and Supplementary Figure 3B; *P* < 0.0001). There was no net effect of IL‐23 on IFNγ (Figure 4E and F). Removing TGFβ increased both GM‐CSF and IFNγ release by mean ± SEM 61% ± 20% (Figure 4C; *P* < 0.01) and mean ± SEM 122% ± 34% (Figure 4E; *P* < 0.01) respectively. IL‐23 cooperated with TGF‐β as removal of both cytokines increased GM‐CSF release by mean ± SEM 185% ± 52% (Figure 4C; *P* < 0.01) and GM‐CSF^pos^ cells by mean ± SEM 128% ± 37% (Figure 4D and Supplementary Figure 3B; *P* < 0.01).

Given the marked reduction in IL‐17^pos^ cells on removing TGFβ, cytokine coexpression was only examined in the presence or absence of IL‐23. There was an overall decrease in the frequency of IL‐17^pos^ Th_17_ (Figure 4B), but an increase in GM‐CSF^pos^ Th_17_ cells (Figure 4D) on removing IL‐23. This corresponded to a proportional increase in polyfunctional IL‐17^pos^ T cells coexpressing especially GM‐CSF (Figure 4G; mean ± SEM 32% ± 3.2% without IL‐23 vs mean ± SEM 41% ± 2.6% with IL‐23; *P* < 0.01).

Th_1_‐polarized cells did not produce IL‐17, and the levels of IFNγ and GM‐CSF were significantly higher (Supplementary Figure 4) than for Th_17_‐polarized CD4 T cells (Supplementary Figure 3). IFNγ release by Th_1_‐polarized cells was 10‐fold that for Th_17_ polarization (mean ± SEM 344 ± 52.4 ng/mL for Th_1_ vs mean ± SEM 33 ± 12.1 ng/mL for Th_17_; *P* < 0.0001), and three‐fold higher for GM‐CSF (5,016 ± 778 pg/mL for Th_1_ vs 1,627 ± 260 pg/mL for Th_17_; *P* < 0.001). Similarly, the percentage of cells producing IFNγ (mean ± SEM 71% ± 1.6% for Th_1_ vs mean ± SEM 20% ± 3.0% for Th_17_; *P* < 0.0001) or GM‐CSF (mean ± SEM 51% ± 5.1% for Th_1_ vs mean ± SEM 22% ± 2.7% for Th_17_; *P* < 0.0001) was higher with Th_1_‐polarizing conditions. The presence of IL‐23 during Th_1_ differentiation reduced GM‐CSF release (Figure 4C, *P* < 0.001) and the frequency of GM‐CSF^pos^ cells (Figure 4D, Supplementary Figure 4A vs B, middle panel; mean ± SEM 51% ± 5.1% minus IL‐23 vs mean ± SEM 18% ± 3.1% plus IL‐23; *P* < 0.001) but did not affect IFNγ (Figure 4E and F). We therefore confirm that IL‐17 production by adaptive immune cells is only partially IL‐23‐dependent in patients with PsA, and IL‐23 cooperates with TGFβ to augment IL‐17 release. Conversely, the presence of IL‐23 reduces GM‐CSF release by Th_17_‐derived and classic Th_1_ cells, in addition to polyfunctional IL‐17^pos^ T cells.

### Polyfunctional Th_17_ and Th_17_‐derived cells are amplified by metabolic stress

The effect of metabolic stress using Th_17_ or Th_1_‐polarizing conditions was evaluated in patients with PsA. Metabolic stress was induced by (1) limiting glycolysis with 2‐DG, (2) exposure to high‐salt medium (ionic stress), or (3) stimulation of ER stress with thapsigargin,^32^ and supernatants harvested for quantification by ELISA. Harvested CD4 T cells were restimulated with PMA and ionomycin, and cytokine release was evaluated by flow cytometry.

**Figure 5:**
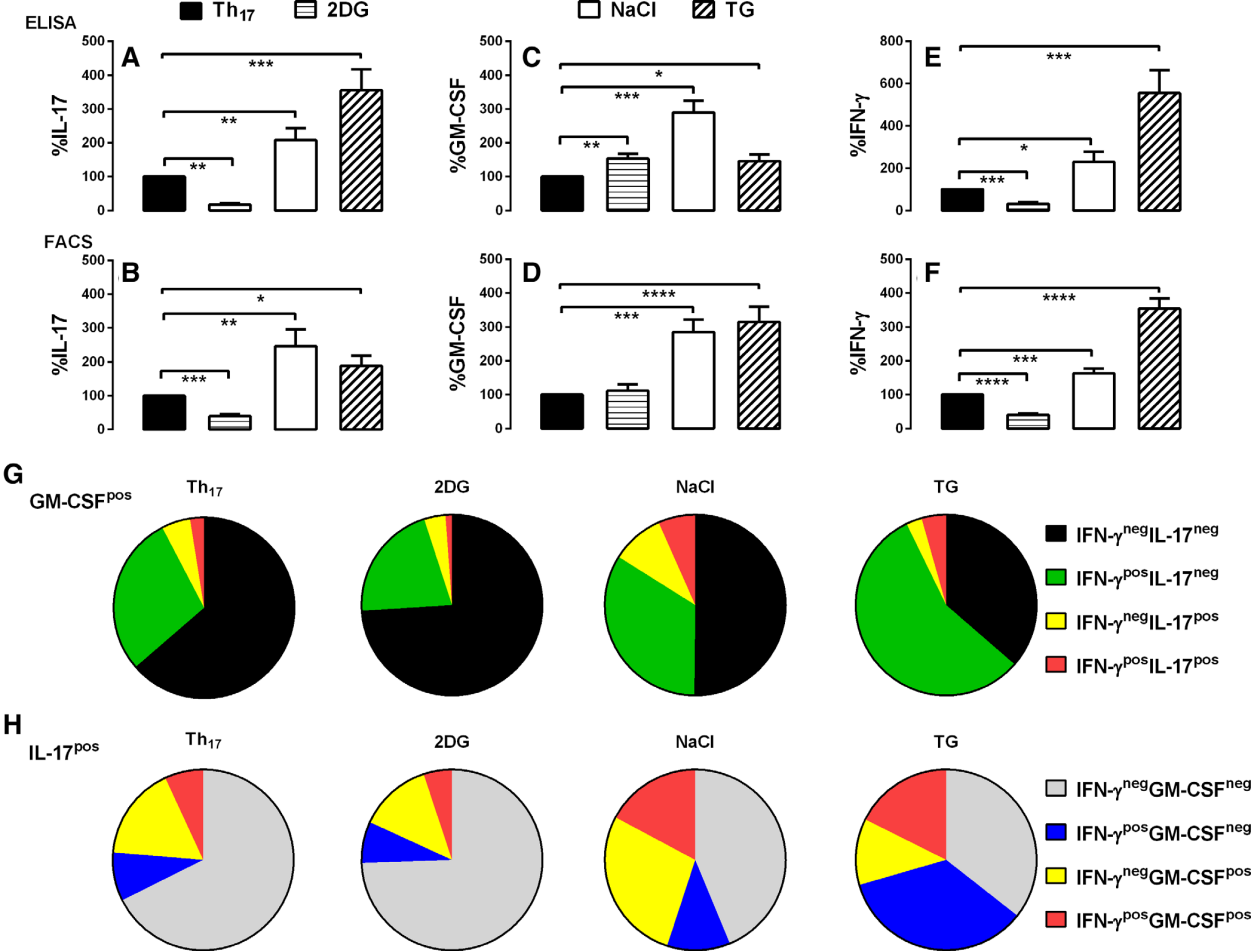
Polyfunctional Th_17_ and Th_17_‐derived cells are amplified by metabolic stress. Naive CD4 T cells from patients with PsA were differentiated in Th_17_‐polarizing conditions in the absence (Th_17_) or presence of the metabolic mediators 2‐DG, NaCl, or TG and stimulated with anti‐CD3/28 Dynabeads for five to seven days, and supernatants were tested for (A) IL‐17, (C) GM‐CSF, or (E) IFNγ by ELISA (normalized to 100% for Th_17_ cells without metabolic mediators). Cells were then restimulated with PMA and ionomycin, before staining and analysis by flow cytometry. (B, D, and E) Percent change in proportion of single cytokine^pos^ cells (normalized to 100% for Th_17_ cells without metabolic mediators) and analysis by FACS for (B) IL‐17, (D) GM‐CSF, or (F) IFNγ. (G) GM‐CSF^pos^ cells coexpressing IFNγ ± IL‐17 were displayed in pie charts as Th_17_ without metabolic mediators, or in the presence of 2‐DG, NaCl, or TG. (H) IL‐17^pos^ cells coexpressing IFNγ ± GM‐CSF were displayed in pie charts as Th_17_ without metabolic mediators, or in the presence of 2‐DG, NaCl, or TG. Data are represented as mean ± SEM, with data analyzed by paired *t‐*test using Tukey's multiple comparison test. *P < 0.05; **P < 0.01; ***P < 0.001; ****P < 0.0001. 2‐DG, 2‐deoxy‐d‐glucose; ELISA, enzyme‐linked immunosorbent assay; FACS, fluorescence‐activated cell sorting; GM‐CSF, granulocyte–macrophage colony‐stimulating factor; IFN, interferon; IL, interleukin; pos, positive; PsA, psoriatic arthritis; TG, thapsigargin; TGF, transforming growth factor. Color figure can be viewed in the online issue, which is available at http://onlinelibrary.wiley.com/doi/10.1002/art.43095/abstract.

Ionic stress with high salt and ER stress with thapsigargin increased IL‐17 (Figure 5A and B, Supplementary Figure 5A), GM‐CSF (Figure 5C and D, Supplementary Figure 5B), and IFNγ (Figure 5E and F, Supplementary Figure 5C) under Th_17_ polarizing conditions. 2‐DG slightly increased GM‐CSF (Figure 5C and D) but inhibited IL‐17 (Figure 5A and B) and IFNγ (Figure 5E and F). 2‐DG correspondingly decreased GM‐CSF^pos^ polyfunctional cells expressing IFNγ ± IL‐17 and IL‐17^pos^ polyfunctional T cells. Thapsigargin was most effective at expanding polyfunctional subsets in which GM‐CSF^pos^ polyfunctional Th_17_‐derived cells increased from mean ± SEM 36% ± 3.4% to 63% ± 3.4% (Figure 5G; *P* < 0.0001), and IL‐17^pos^ polyfunctional Th_17_ from mean ± SEM 32% ± 3.2% to 65% ± 3.1% (Figure 5H; *P* < 0.0001). Thapsigargin preferentially expanded IFNγ expression in GM‐CSF^pos^ (Figure 5G) and IL‐17^pos^ (Figure 5H) polyfunctional Th_17_, whereas high salt augmented IL‐17^pos^ cells coexpressing GM‐CSF (Figure 5H; mean ± SD 24% ± 3.2% to 45% ± 4.7%; *P* < 0.001).

As observed for Th_17_‐polarized cells, IFNγ frequency within Th_1_‐differentiated cells was reduced with 2‐DG (Supplementary Figure 6; mean ± SEM 34% ± 2% decrease for Th_1_ vs mean ± SEM 60% ± 5% for Th_17_) whereas there was no significant effect on GM‐CSF (Supplementary Figure 6A, C, and D). There was a modest increase in IFNγ (mean ± SEM 18% ± 5% increase for Th_1_ vs mean ± SEM 254% ± 30% for Th_17_) and GM‐CSF (mean ± SEM 33% ± 21% increase for Th_1_ vs mean ± SEM 215% ± 44% for Th_17_) with thapsigargin. High salt did not expand Th_1_‐differentiated cells (Supplementary Figure 6), whereas a decrease in cell viability was observed in some donors (Supplementary Figure 6A and B), although no overall net decrease in cytokine release was observed for pooled donors (Supplementary Figure 6C and D).

We therefore demonstrate that metabolic stress expands putatively pathogenic polyfunctional Th_17_ and Th_17_‐derived cells but minimally affects Th_1_ subsets. We also reveal important differences in the regulation of IL‐17 and GM‐CSF, including differential effects on limiting glycolysis as well as reciprocal regulation by IL‐23. The cytokine milieu and metabolic microenvironment experienced during the expansion of naive CD4 T cells will likely influence the T cell phenotypes obtained, in which there is considerable Th_17_ plasticity, and such factors may be important in maintaining these subsets at sites of disease.

## DISCUSSION

We demonstrate increased proportions of GM‐CSF^pos^ Th_17_‐derived and IL‐17^pos^ polyfunctional T cells in the inflamed joints of patients with PsA, which are reduced in frequency in the PB of patients with PsA when compared with PB from HDs. Polyfunctional T cells may traffic to inflamed joints and are retained or expanded within the joint. IL‐17^pos^ T cells are by definition Th_17_, in which the coexpression of pro‐inflammatory GM‐CSF implies a pathogenic phenotype.^11^, ^12^, ^13^, ^14^, ^15^ These polyfunctional Th_17_ cells coexist with GM‐CSF^pos^ cells no longer producing IL‐17 but demonstrating features of Th_17_ (CCR6^pos^, CD161^pos^), described as Th_17_‐derived, ex‐Th_17_, or nonclassic Th_1_ cells. The pathogenicity of Th_17_‐derived cells or the switch of Th_17_ to Th_1_ correlates with expression of T‐bet.^15^, ^16^, ^17^ In contrast, nonpathogenic Th_17_ cells that play a role in barrier function in the intestine coproduce anti‐inflammatory mediators such as IL‐10, have an ex‐Th_17_ regulatory phenotype, and are not influenced by IL‐23.^33^ Th_17_ cells therefore exhibit considerable plasticity and may transform to a Th_17_‐derived phenotype at sites of disease.^34^ Th_17_‐derived cells have a more differentiated phenotype, enhanced survival capability, increased proliferative capacity, and are polyfunctional in terms of cytokine production relative to classic Th_1_ and Th_17_ cells, suggesting they are more likely to contribute to disease pathogenesis.^34^

We confirm IL‐23–dependent, but also IL‐23–independent IL‐17 production by adaptive immune cells, in which the absence of IL‐23 during Th_17_ differentiation reduced IL‐17 by mean ± SEM 31% ± 5.8%. In a Th_17_ fate reporter mouse model, chronic inflammation caused a switch to alternative cytokines by Th_17_ cells that no longer produced IL‐17 (Th_17_‐derived cells), for which this conversion was IL‐23 dependent.^17^ IL‐23 also drove pathogenic T cell responses and reduced regulatory T cell activity in intestinal inflammation, whereas IL‐23R activation induced T‐bet signaling and the generation of pathogenic polyfunctional T cells expressing IL‐17A with IFNγ.^35^, ^36^ In contrast to murine studies in which IL‐23 augments GM‐CSF,^11^, ^12^ we show that GM‐CSF is negatively regulated by IL‐23 in human Th_17_ and Th_1_ cells. Removing IL‐23 during Th_17_ differentiation increased the frequency of polyfunctional IL‐17^pos^ T cells coexpressing GM‐CSF. There are therefore key differences in murine and human Th_17_ cells and IL‐23‐responsiveness, and it is plausible that augmentation of GM‐CSF when blocking IL‐23 could be a factor determining differences observed between IL‐17 and IL‐23 inhibition in human disease.^10^ We also confirmed dependence on TGFβ for IL‐17 release by human naive CD4 T cells differentiated under Th_17_‐polarizing conditions.^37^ TGFβ cooperated with IL‐23 in our study to enhance IL‐17 production. TGFβ therefore appears to be an essential cytokine for human Th_17_ differentiation, although previous studies have shown that other pro‐inflammatory mediators including IL‐1β, IL‐6, and IL‐23 are required to confer pathogenicity.^22^, ^23^, ^24^, ^25^, ^26^, ^27^, ^38^ The plasticity of Th_17_ cells and the associated cytokine milieu means that targeting of IL‐17 alone will only transiently inhibit Th_17_ or Th_17_‐derived cells in chronic inflammatory settings.

We show that both ionic pressure with high salt and ER stress with thapsigargin expand polyfunctional Th_17_‐derived and IL‐17^pos^ Th_17_ cells in vitro, in accordance with previous murine data.^32^ Limiting glycolysis with 2‐DG can increase IL‐17,^32^ whereas we showed that 2‐DG inhibited IL‐17 and IFNγ, but increased GM‐CSF. Shi et al^39^ also demonstrated that blocking glycolysis with 2‐DG inhibited Th_17_ development, in which the dichotomy of 2‐DG is likely influenced by glucose availability and in particular the expression of the glycolytic intermediate metabolite, phosphoenolpyruvate.^40^ There is further in vivo evidence to support metabolic stress as a mediator of Th_17_ expansion, in which a high‐salt diet fed to mice increased the severity of EAE, whereas serum glucocorticoid kinase (SGK) 1 deficient mice exhibited less severe EAE.^28^, ^29^ SGK1 governed sodium transport, salt homeostasis, IL‐23R expression, and stabilization of the Th_17_ phenotype, thus linking metabolic and inflammatory pathways. Furthermore, mice fed a high‐salt diet had an altered microbiome in which *Lactobacillus murinus* was depleted, and *L murinus* treatment prevented salt‐induced exacerbation of murine EAE by modulating Th_17_ cells.^30^ A human study of moderate salt challenge also showed reduced survival of *Lactobacilli spp*, with a concomitant increase in Th_17_ and blood pressure.^30^ In a murine model of collagen‐induced arthritis, clinical and histologic arthritis were more severe in mice fed a high‐salt diet, the expression of intestinal and synovial IL‐17 was higher, and higher salt levels were demonstrated in mice with RA when compared with the SF from mice with osteoarthritis.^41^

The impact of ionic stress on Th_17_ expansion is influenced by the cytokine microenvironment. Matthias et al^42^ showed that high salt exposure induced several anti‐inflammatory mediators in Th_17_ cells, including the regulatory transcription factor FOXP3, TGFβ, IL‐10, and CTLA‐4. However, the presence of pro‐inflammatory cytokines prevented the upregulation of anti‐inflammatory mediators in human Th_17_ cells, and in murine EAE, high salt was only pathogenic when Th_17_ cells were primed in the presence of pro‐inflammatory mediators (IL‐6, IL‐1β, IL‐23) and absence of TGFβ.^42^ Brucklacher‐Waldert et al^32^ confirmed a requirement for pro‐inflammatory mediators to augment IL‐17 release with thapsigargin and high salt, but in contrast with Matthias et al,^42^ TGFβ increased IL‐17 release elicited by high salt. Overall, these data highlight the complex interaction of cytokine and metabolic microenvironments in influencing the pro‐ versus anti‐inflammatory Th_17_ subsets that ensue.

Matthias et al^42^, ^43^, ^44^ reported that high salt augmented IL‐17 but with a concomitant reduction in IFNγ and shift from Th_1_ to Th_2_ responses. In our study, we showed the effect of salt on IFNγ was context‐dependent in that high salt augmented IFNγ and GM‐CSF release in Th_17_‐polarizing but not Th_1_‐polarizing conditions. TGFβ is an inducer of SGK1,^29^ and NaCl can increase the production of TGFβ by NaCl‐induced NFAT5,^42^, ^45^ demonstrating an autocrine effect of TGFβ on Th_17_ cells, and positive relationship with NaCl. A putative candidate is the relationship between SGK1 and IL‐23 downstream signaling, in which SGK1 stabilizes IL‐23R expression and the Th_17_ phenotype by deactivation of FOXO1, a direct repressor of IL‐23R expression.^29^ Collectively, these studies provide a direct molecular link between Th_17_ immunity and ionic stress that may be crucial to the development and expansion of pathogenic Th_17_ subsets.

The ER is an organelle that facilitates protein folding, trafficking, and degradation to maintain protein homeostasis, and ER stress agents such as thapsigargin disturb ER proteostasis, thus activating the unfolded protein response. There are several physiologic mediators of ER stress including hypoxia, in which XBP1 is a downstream transcription factor activated by ER stress, which directly regulates *Rorc* and thus Th_17_ cell differentiation. XBP1 also forms a transcriptional complex with hypoxia‐inducible factor (HIF) 1α, a metabolic sensor of hypoxia and a key transcriptional regulator of Th_17_ cell polarization operating via direct transcriptional activation of *Rorc*.^39^, ^46^, ^47^ The potent augmentation of GM‐CSF^pos^ Th_17_‐derived and IL‐17^pos^ polyfunctional Th_17_ cells by thapsigargin identifies the ER stress response as a putative molecular target. Subudhi et al^48^ showed metabolic coordination between skin epithelium and type 17 immunity in plaque psoriasis, in which blocking HIF1α in psoriatic lesions ex vivo impaired glycolysis and phenocopied IL‐17 inhibition.^48^ They showed that epithelial pathology was fueled by glycolysis to enhance lactate production, whereas pharmacological inhibition of lactate‐producing enzymes or lactate transporters attenuated skin pathology and IL‐17A expression in vivo, linking metabolic stress and type 17 immunity for patients with psoriatic disease.^48^ Lactate accumulation occurs in metabolically active inflamed joints, and uptake of lactate by the SLC5A12 transporter into CD4 T cells augments IL‐17 production.^49^

A limitation of our study was the number of phenotypic markers and intracellular cytokines used to characterize the defined T cell subsets in contrast to more recent studies in patients with PsA. Steel et al^8^ described an abundance of polyfunctional IL‐17A^pos^CD8^pos^ SF T (Tc_17_) cells, possessing features of Th_17_ cells (expressing *RORC/IL23R/CCR6/CD161*) and Tc1 (granzyme A/B). These Tc_17_ cells demonstrated a tissue‐resident memory (T_RM_) cell signature and had increased levels of CXCR6 hypothesized to retain the subset within the joint by binding to the ligand CXCL16; the latter also increased in the synovial joints of patients with PsA. Penvaka et al^50^ described clonally expanded CD8^pos^ T cells in SF from patients with PsA, with abundant expression of CXCR3, in which CXCR3 plays a role in chemotaxis during inflammation. Polyfunctional T cells within our study likely include T_RM_ cells, in which approximately 50% of GM‐CSF^pos^ CD4 and CD8 SF cells coexpressed CD69, compared with 20% of corresponding PB cells (data not shown because they have not extensively been characterized).

In summary, we hypothesize that the abundance polyfunctional GM‐CSF^pos^ and IL‐17^pos^ type 17 cells in the SF of patients with PsA represents the appearance or accumulation in the joint of Th_17_‐derived subsets (or nonclassic Th_1_) that express Th_17_ cell‐associated markers. IL‐23 augments the frequency of IL‐17^pos^ cells, but there is probable IL‐23–independent Th_17_ differentiation and IL‐17 release by adaptive immune cells. In contrast to murine studies, IL‐23 negatively regulates GM‐CSF in human T cells. TGFβ is required for Th_17_ differentiation and cooperates with IL‐23 to augment IL‐17 production. IL‐17^pos^ Th_17_ and GM‐CSF^pos^ Th_17_‐derived polyfunctional T cell subsets, but not classic Th_1_ cells, are expanded by metabolic stress, for which there is accumulating evidence linking metabolic stress with type 17 immunity. Our data therefore support GM‐CSF inhibition and/or modulation of metabolic stress as novel therapeutic targets for patients with PsA.

## AUTHOR CONTRIBUTIONS

All authors contributed to at least one of the following manuscript preparation roles: conceptualization AND/OR methodology, software, investigation, formal analysis, data curation, visualization, and validation AND drafting or reviewing/editing the final draft. As corresponding author, Dr Stober confirms that all authors have provided the final approval of the version to be published, and takes responsibility for the affirmations regarding article submission (eg, not under consideration by another journal), the integrity of the data presented, and the statements regarding compliance with institutional review board/Declaration of Helsinki requirements.

## Supporting information


**Appendix S1.** Supporting Information
