# Supplementary material for: Metabolic Stress Expands Polyfunctional, Proinflammatory Th17 Cells in Patients With Psoriatic Arthritis for Whom There is Interleukin‐23–Independent Interleukin‐17 Production
Source: Arthritis Rheumatol. 2025 Feb 8;77(7):842–53. doi: 10.1002/art.43095 (PMC12209755; doi:10.1002/art.43095)
Supplement: Supplementary file 1 — Appendix S1. Supporting Information [file ART-77-842-s001.docx]

**Supplementary Data**

***Figure S1:* Purity of naïve CD4 T cells following isolation of untouched cells using the Naïve CD4 T cell isolation kit II human**

**
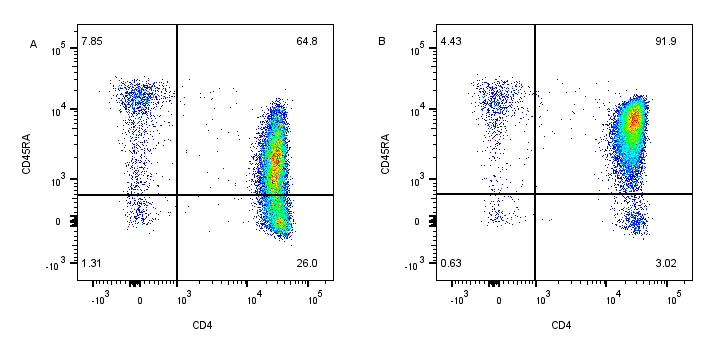
**

**
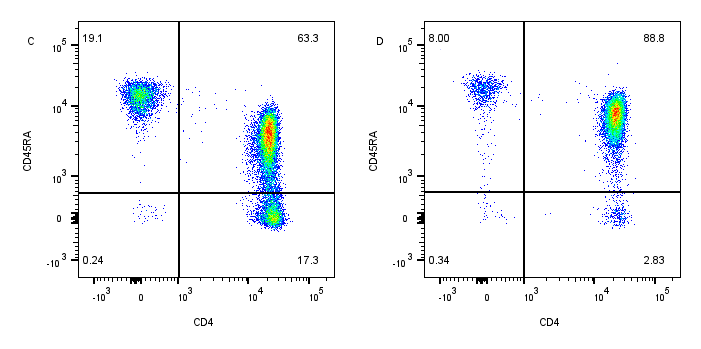
**

PBMC were purified by density centrifugation (6). Naïve CD4 T cells were isolated using the Naïve CD4 T cell isolation kit II human (Miltenyi Biotec). This method depletes memory CD4 and non-CD4 T cells from PBMC by using a cocktail of biotinylated antibodies, and these labelled cells are removed with anti-biotin microbeads. Two typical PsA patient samples of isolated untouched naïve CD4 T cells are shown in the right panels (B, D). The left panels A, C show the corresponding eluted biotinylated fractions. CD4+CD45RA^pos^ were generally >80%, where the majority of CD4 cells were CD45RA^pos^, with a small proportion of CD4^neg^CD45RA^pos^ cells as demonstrated

***Figure S2:*** **Polyfunctional GM-CSF^pos^ and IL-17^pos^ T cells are abundant in PsA inflamed joints, and are decreased in PsA peripheral blood**

**
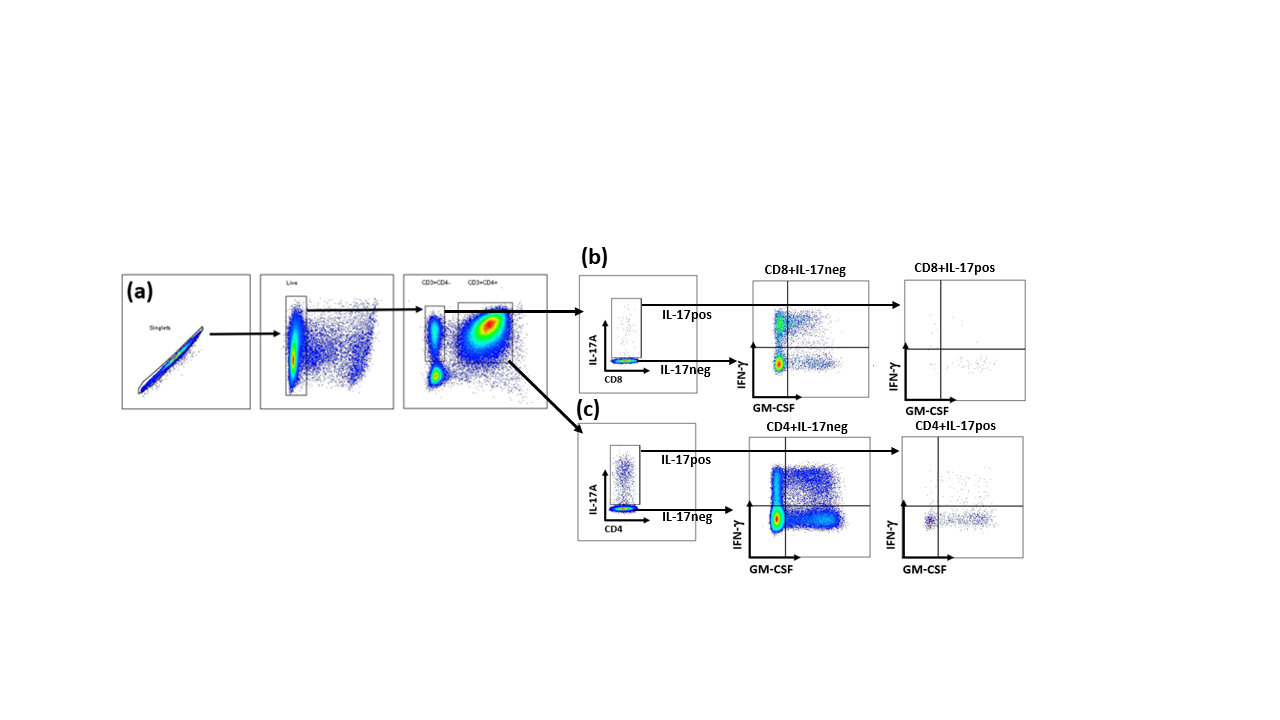
**

**

**

Matched donor PsA peripheral blood (PB) (n=4) or PsA synovial fluid (SF) (n=4) PBMC or SFMC were stimulated with PMA and ionomycin, prior to staining and analysis by flow cytometry. PBMC and SFMC were gated on FSC/SSC to identify lymphocytes; doublets and dead cells excluded; and further gated as CD3+CD4^neg^ (CD8) or CD3+CD4+ (CD4) T cells. Live, singlet CD8 (b, upper panel) or CD4 (c, lower panel) T cells were gated as IL-17 negative (left panel) or IL-17 positive (right panel), and then gated on IFN-γ and GM-CSF expression.

Figs d,e demonstrate decreased frequencies of GM-CSF^pos^ (IFN-γ^neg^ IL-17^neg^) (d) CD8 and (e) CD4 T cells in matched patient PsA patient SF (■) relative to PB (▲). Figs f,g show increased frequencies of polyfunctional type 17 IL-17^pos^GM-CSF^pos^IFN-γ^pos^ (f) CD8 and (g) CD4 T cells in matched patient PsA patient SF (■) compared to PB (▲).

***Figure S3:*** **IL-17, GM-CSF and IFN-γ release by Naïve T cells differentiated under Th_17_-polarising conditions in the presence or absence of IL-23 and TGF-β**

**
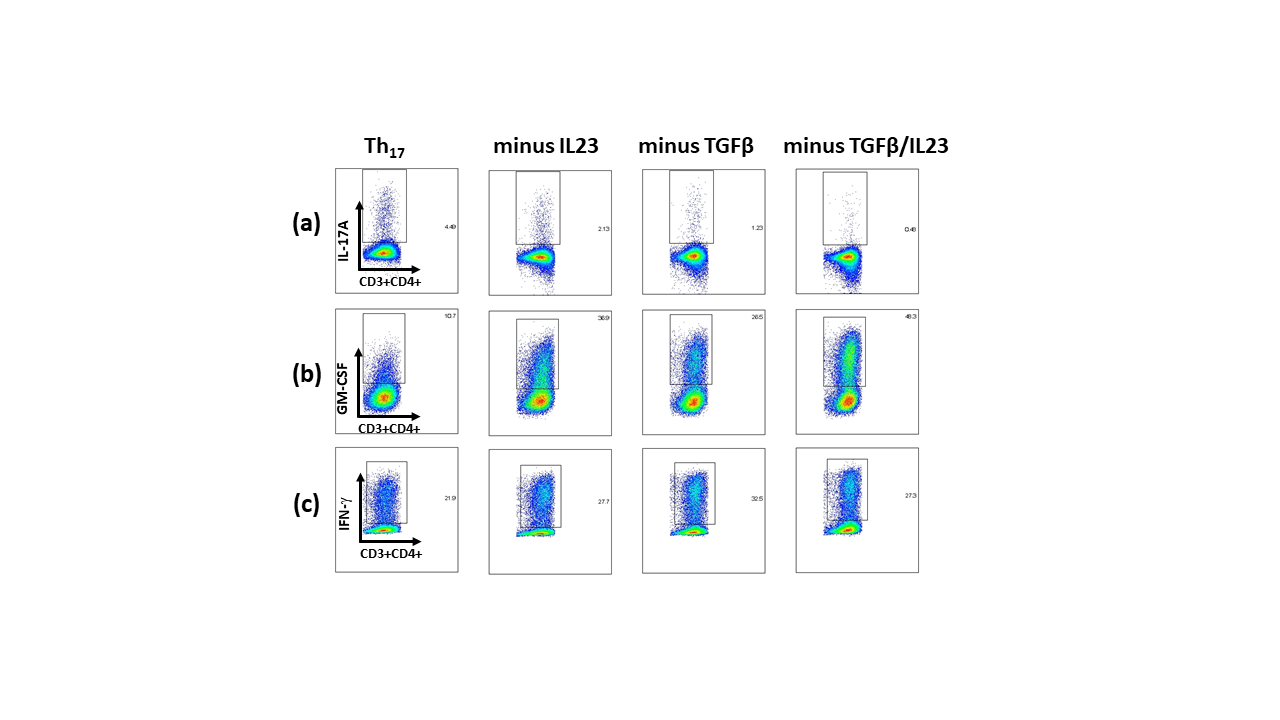
**

PsA patient naïve CD4 T cells were differentiated under Th_17_-polarising conditions (Th_17_, left panel) in the presence of IL-1β, IL-6, anti-IFN-γ, IL-23 and TGF-β. Th_17_ differentiation was performed under the same conditions with the removal of IL-23 alone (minus IL23); TGF-β alone (minus TGFβ); or on removing IL-23 and TGF-β (minus TGFβ/IL23). Cells were stimulated with anti-CD3/28 Dynabeads for 5-7 days. Cells were then harvested, re-stimulated with PMA and ionomycin, prior to staining and analysis by flow cytometry. Cells were gated as live, CD3+CD4+ T cells, and evaluated as (a) IL-17^pos^, (b) GM-CSF^pos^, or (c) IFN-γ^pos^ T cells, in the absence or presence of IL-23 ± TGF-β as detailed. A representative single PsA patient sample is shown by flow cytometry

***Figure S4:* IFN-γ and GM-CSF release by Naïve T cells differentiated under Th_1_-polarising conditions in the presence or absence of IL-23**

**
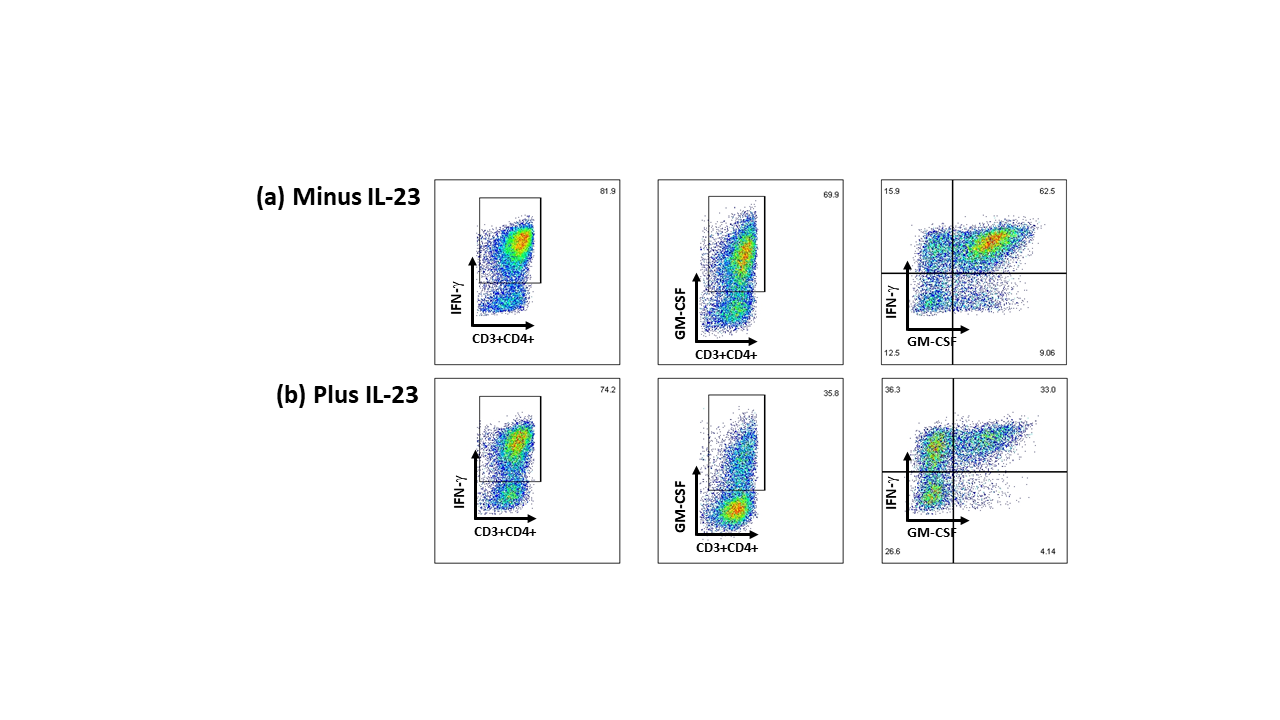
**

PsA patient naïve CD4+ T cells were differentiated under Th_1_-polarising in the presence of (a) IL-12 (minus IL-23), or (b) IL-12 plus IL-23 (plus IL-23). Cells were stimulated with anti-CD3/28 Dynabeads for 5-7 days. Cells were then harvested, re-stimulated with PMA and ionomycin, prior to staining and analysis by flow cytometry. Cells were gated as live, CD3+CD4+ T cells, and evaluated as IFN-γ^pos^ (left panel), GM-CSF^pos^ (middle panel), or for co-expression of IFN-γ with GM-CSF (right panel). A representative single PsA patient sample is shown by flow cytometry

***Figure S5:* IL-17, GM-CSF and IFN-γ release by Naïve T cells differentiated under Th_17_-polarising conditions in the presence or absence of metabolic stress**

**
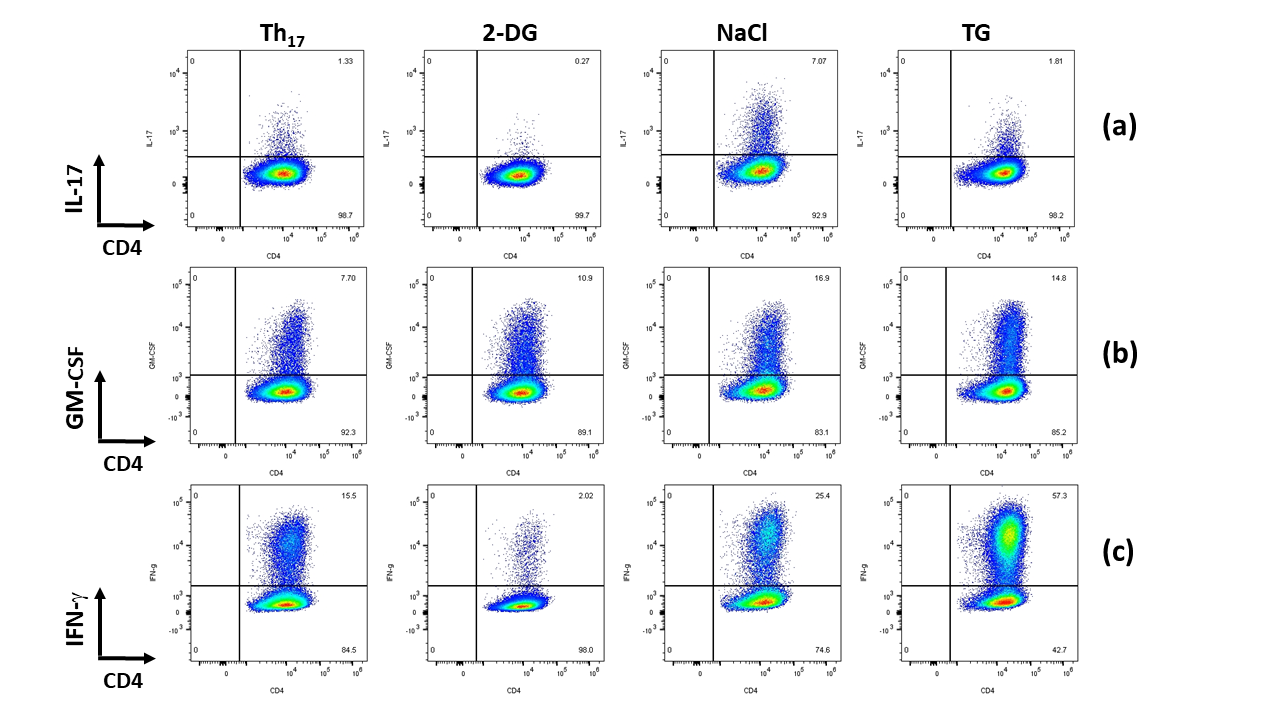
**

PsA patient naïve CD4+ T cells were differentiated under Th_17_-polarising conditions (Th_17_, left panel, plus IL-1β, IL-6, anti-IFN-γ, IL-23, TGF-β) with the addition of the metabolic mediators 2-DG, NaCl or Thapsigargin (TG). Cells were stimulated under these conditions with anti-CD3/28 Dynabeads for 5-7 days. Cells were then harvested, re-stimulated with PMA and ionomycin, prior to staining and analysis by flow cytometry. Cells were gated as live, CD3+CD4+ T cells, and evaluated as (a) IL-17^pos^, (b) GM-CSF^pos^, or (c) IFN-γ^pos^ T cells, in the absence or presence of 2-DG, NaCl or TG as detailed. A representative single PsA patient sample is demonstrated by flow cytometry

***Figure S6:* GM-CSF and IFN-γ release by Naïve T cells differentiated under Th_1_-polarising conditions in the presence or absence of metabolic stress**

**
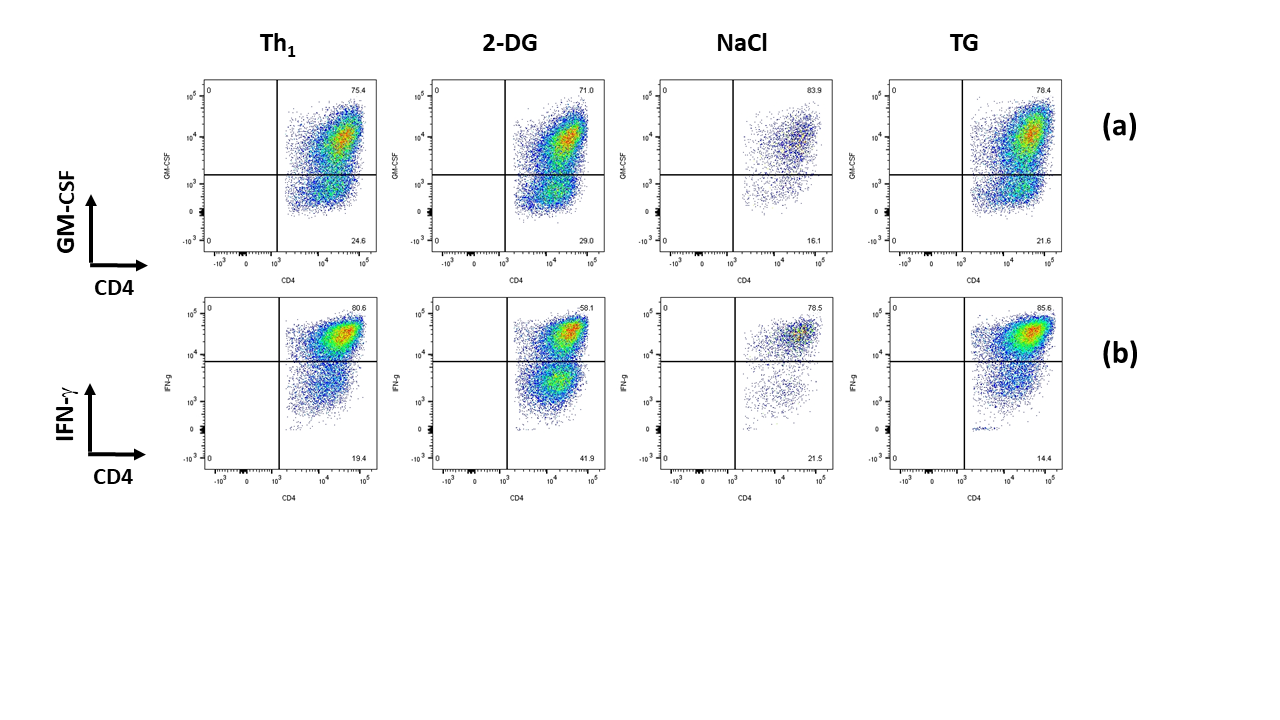
**

**

**

PsA patient naïve CD4+ T cells were differentiated under Th_1_-polarising conditions with the addition of the metabolic mediators 2-DG, NaCl or Thapsigargin (TG) (n=10). Cells were stimulated under these conditions with anti-CD3/28 Dynabeads for 5-7 days. (c) Supernatants were removed for evaluation of GM-CSF or IFN-γ by ELISA. Additionally, cells were harvested, re-stimulated with PMA and ionomycin, prior to staining and analysis by flow cytometry. Cells were gated as live, CD3+CD4+ T cells, and evaluated as (a) GM-CSF^pos^, or (b) IFN-γ^pos^ T cells, in the absence or presence of 2-DG, NaCl or TG as detailed, with a representative PsA patient sample demonstrated by flow cytometry. (d) Flow cytometry data is represented as the percentage change in the proportion of single cytokine^pos^ cells (normalised to 100% for Th_1_ cells without metabolic mediators), and analysed as CD4+ cells producing GM-CSF (left panel) or IFN-γ (right panel).
